# Supervision programs implemented by nurses to caregivers in community: a scoping review protocol

**DOI:** 10.3389/fpubh.2025.1473912

**Published:** 2025-03-25

**Authors:** Inês Moreira, Márcia Coelho, Mauro Mota, Regina Pires, Margarida Reis Santos

**Affiliations:** ^1^Nursing School of Porto, Porto, Portugal; ^2^Local Health Unit of the Aveiro Region, Aveiro, Portugal; ^3^CINTESIS@RISE (Center for Health Technology and Services Research), Porto, Portugal; ^4^Health Sciences Research Unit: Nursing (UICISA), Coimbra, Portugal; ^5^Viseu Higher School of Health, Viseu, Portugal; ^6^Institute of Biomedical Sciences Abel Salazar, University of Porto, Porto, Portugal

**Keywords:** caregiver, supervisory program, clinical supervision, nurse, community caregiver, community

## Abstract

**Introduction:**

With the increase in the number of informal caregivers, their training becomes essential. The supervisory programs implemented by nurses play a leading role in caregiver training.

**Objectives:**

To map the supervisory programs implemented by nurses to caregivers in the community.

**Methods:**

A scoping review will be developed according to the guidelines of the Joanna Briggs Institute. Studies published and unpublished in English, Portuguese or Spanish since 1993 will be considered. The results of the research, study selection and inclusion process will be presented in a PRISMA flowchart for scoping reviews.

**Results:**

Mapping the evidence will allow us to analyze the supervision programs implemented by nurses for caregivers in the community. The results will be presented through an extraction table according to the objectives.

**Conclusion:**

This review is expected to constitute a starting point for the critical analysis of studies relating to supervisory programs implemented by nurses for caregivers.

## 1 Introduction

Increasing average life expectancy and chronic diseases have increased the demand for long-term healthcare and the need for informal caregivers (1). In 2019, more than a fifth (20.3%) of the European Union population was aged 65 or over. It is projected that there will be close to half a million centenarians in the EU-27 by 2050 (2). Current statistics indicate that between 2018 and 2080, the aging rate will approximately double, and for every 100 young people there will be 300 older adult people (2). According to the National Alliance for Caregiving and Real Possibilities Public Policy Institute, it is expected that by 2030 the number of caregivers will reach 21.5 million and that the time of care will be at least 20 h per week (3).

There are two main types of care for seniors who are aging and need assistance with their care. The first is formal care, which generally refers to paid care offered by medical institutions or trained health professionals to those in need (4). The second type is informal care, which covers unpaid assistance provided by family, close relatives, friends and neighbors. Both formal and informal care involve a variety of activities, but informal caregivers rarely receive adequate training to perform these functions (4).

According to the Family Caregiver Alliance (5), an informal caregiver is any family member, neighbor or friend who has a significant personal relationship and who is able to meet the needs of the older adult person or adult with a chronic or disabling condition. These people may be primary or secondary caregivers and may or may not live with a person receiving care.

More than 300 million residents of the European Union are aged between 18 and 64 and around a third of this population works as caregivers. The percentage of caregivers who only have care responsibilities for incapacitated family members amounts to 12% (6). Data from some countries show that the majority of informal caregivers take care of their parents (51.4%), followed by those who take care of their spouses (18%) and children (12.7%). In addition to the transformation in personal and family life habits, it has also been equitably observed that caregivers have physical, psychological and social needs and also feel the need for information, training and professional support (3).

The multiplicity of tasks performed daily in supporting people in situations of severe dependency translates into levels of intense overload, which has a negative impact on the caregiver’s life, particularly on their health, work, and social activities that are detrimental to their well-being (7). The provision of guidance and support by health and nursing professionals, in order to improve the skills of older adult caregivers, also results in lower levels of overload, physical and emotional exhaustion and, consequently, a better quality of life (8). In the process of transition of the caregiver to the exercise of the role, it’s necessary to acquire and develop skills, namely cognitive, related to knowledge, mastery, inherent to instrumental skills, and knowing how to be, intrinsic to personal development (9). As nurses are the health professionals who have the closest contact with people, families and the community, they act as a preponderant factor in health outcomes (2).

Over the last few years, the care provided by caregivers has been a concern for nurses and consequently for clinical supervision (10). Clinical supervision plays a leading role in the training of caregivers. This is defined as formal process of professional support and learning that provided the development of knowledge and skills in order to take responsibility for one’s own practice, improving the protection of supervisees and the safety of care (11). Over the years, clinical supervision of nurses has been considered a mechanism to promote the quality and safety of care (12). Clinical supervision also plays a preponderant role in the management of health organizations in the sense that it allows the head nurse, as a manager, to develop strategies that promote the quality of care, with direct repercussions on health gains (13).

Training the caregiver implies providing them with knowledge that makes it possible for them to have a better appreciation of themselves, to be able to recognize in themselves their physical and emotional capacities to deal with this new reality, thus arising the need to implement supervisory programs as a way to ensure the training of caregivers (14). Training informal caregivers should be seen as a safe way to obtain better results in rehabilitation, higher levels of autonomy and reduced costs and readmissions (15).

A program can be defined as a systematic and deliberate intervention that results from the identification of the needs of a given population or group, is objective-oriented, and is grounded with theory that will give rigor and credibility to the action. The intervention aims to meet the needs of the recipients (16). An intervention program addresses a set of needs and develops the skills required for positive change. For this reason, a program-based intervention requires a set of actions and resources that are designed, applied and evaluated, in an organized manner, in a given social reality and following a sequence of specific stages (2).

Considering the evidence found on the subject, it is perceived that it is insufficient and unclear, and it is appropriate to carry out a scoping review to map all the supervisory programs implemented by nurses to caregivers in the community. Knowledge about how supervisory programs are implemented by nurses for caregivers is still scarce. Therefore, this lack of knowledge is the reason why the applicability and potential effectiveness of caregiver-provided care is still unknown. For this reason, supervisory mapping programs related for clinical nursing supervision is highly relevant. A scoping review is essential to analyze the extent, scope and nature of the research activity. It is also pertinent to map underdeveloped areas of study (17). A previous search was conducted in MEDLINE, Cochrane Database of Systematic Reviews and Open Science Framework (OSF), and no current or ongoing systematic reviews or scoping reviews on the topic were identified.

This scoping review aims to answer the following questions:

1. What are the supervisory programs implemented by the nurse to the caregiver?
2. What are the characteristics of the supervisory programs?
3. In what contexts are supervisory programs applied?

## 2 Methods

A scoping review will be developed and will follow JBI methodology for scoping reviews, while reporting will adhere to PRISMA-ScR guidelines (18) and is registered in the Open Science Framework (https://osf.io/s6wfv/). The review will last 6 months.

### 2.1 Inclusion criteria

In this scoping review, the Participants (P) are informal caregivers who are over 18 years old regardless of their literary qualifications and time as caregivers. These caregivers can be family members, friends or neighbors of the person needing care and are responsible for meeting all the needs of the dependent person (5). Some of the needs that need to be met are self-care needs (hygiene, food, mobility) (19). Caregivers themselves also have some needs, such as social needs that can be met through informal support and financial needs (20).

The Concept (C) under analysis are the supervisory programs implemented by the nurse to the caregiver. A program can be defined as a systematic and deliberate intervention that results from the identification of the needs of a given population or group. A program-based intervention requires a set of actions and resources that are designed, applied and evaluated, in an organized manner, in a given social reality (16). These include informative and educational programs (21, 22) and protocols (23). The programs to be included can be done remotely or in person and will be considered in the study regardless of the frequency and assiduity of being implemented.

The Context (C) of this scoping review is the community. This scoping review will examined research on clinical supervision for nurses in non-hospital settings, with the aim of improving the quality of care provided by caregivers. The review specifically focused on community contexts, which encompass various locations like homes, health centers, day care centers, nursing homes, and residential care facilities, regardless of country or cultural differences (23).

### 2.2 Search strategy

All studies that address the supervisory programs implemented by nurses in the community to caregivers will be analyzed. This review includes studies published in English, Portuguese and Spanish since 1993. Since this year there has been a renewal in the concept, research and implementation of clinical supervision (11).

The search strategy will aim to search for published and unpublished studies. A limited initial search was conducted in MEDLINE (PubMed) and CINAHL (EBSCOhost) to identify literature on the topic. The search strategy will include all identified keywords and index terms and will be adapted to each database and/or information source included (Table 1). The reference list of all included sources of evidence will be evaluated for further studies.

**Table 1:** Search strategy: MEDLINE (PubMed) on February 24th 2024.

Search	Query	Results
#1	(((((caregiver*[Title/Abstract]) OR (informal caregiver[Title/Abstract])) OR (carer*[Title/Abstract])) OR (family caregiver[Title/Abstract])) OR (care-giver[Title/Abstract]))	117,444
#2	(Caregivers[MeSH Terms])	52,130
#3	(((((((program*[Title/Abstract]) OR (programs, training[Title/Abstract])) OR (clinical supervision[Title/Abstract])) OR (supervisory program*[Title/Abstract]))	1,136,100
#4	(Program Development[MeSH Terms])) OR (Nursing, Supervisory[MeSH Terms]))	38,641
#5	(((((((((communit*[Title/Abstract]) OR (domicile[Title/Abstract])) OR (home care[Title/Abstract])) OR (home nursing[Title/Abstract])) OR (residential care home[Title/Abstract])) OR (home help[Title/Abstract]))	804,834
#6	(Home Nursing[MeSH Terms])) OR (Home Care Services[MeSH Terms])) OR (Nursing Homes[MeSH Terms])) OR (Community Support[MeSH Terms])	94,334
#7	#1 OR #2	127,747
#8	#3 OR #4	1,157,562
#9	#5 OR #6	876,395
#10	#7 AND #8 AND #9	6,077
#11	#10 AND Filters: English, Portuguese, Spanish, from 1993 to 2024	5,776

The search will be conducted in several databases of published and unpublished studies: MEDLINE (PubMed), CINAHL (EBSCOhost), PsycINFO (EBSCOhost), Nursing and Allied Health Collection (EBSCOhost), Cochrane Central Register of Controlled Trials (EBSCOhost), MedicLatina (EBSCOhost), Web of Science, Scopus, LILACS, WorldWideScience. This search will also include sources of unpublished studies such as ProQuest Dissertations and Theses and Repositório Científico de Acesso Aberto em Portugal (Open Access Scientific Repository in Portugal – RCAAP), PQDT Open – ProQuest Dissertations and Theses. After the search, all identified citations will be collected and directed to Rayyan – Intelligent Systematic Review (24) and duplicates will be eliminated. Subsequently, the titles and abstracts will be evaluated by two independent reviewers (IM, MSC). Potentially relevant sources will be retrieved in full. The full text of the selected articles will be evaluated in detail against the inclusion criteria by two independent reviewers (IM, MSC). Reasons for exclusion from the scope review will be recorded. Any discrepancies that may arise in any of the stages of the study selection process will be resolved through discussion or using a third reviewer (MM).

The results of the research and the process of inclusion of the studies will be described in full in the final scoping review and presented in a flow diagram Preferred Reporting Items for Systematic Reviews and Meta-analyses extension for scoping review (PRISMA-ScR) (25).

### 2.3 Data extraction

Data extraction from the articles included in the scoping review will be done by two independent reviewers (IM, MSC) using a data extraction tool developed by the reviewers (Table 2). The extracted data will include specific details about the participants, concept, context, study methods and key findings relevant to answering the research questions. During the process of extracting data from each included evidence source, this tool will be modified and revised as needed. Any discrepancies that arise during the extraction of results will be resolved through the third reviewer (MM).

**Table 2:** Data extraction instrument.

Data extraction table in a scoping review	
Author year	
Article title	
Aim	
Study methods	
Supervisions programs implemented and their characteristics	
Context	
Results	
Main conclusions	

All modifications will be detailed in the scoping review. In case of need, the authors of the articles will be contacted in order to request missing data or additional data.

For each research question formulated in this scoping review, tables will be developed with all the respective data obtained. A summary will also be developed with all the results, which will accompany the tabulated and mapped results, to answer and relate the results with the objectives and questions of this scoping review.

## 3 Results

It is expected that the results found demonstrate the importance of applying supervision programs to improve the quality of care provided by caregivers. The application of supervisory programs implemented by nurses is an added value for the caregiver in the sense that it will enhance their training to care, to ensure that all the needs of the dependent person are met and consequently guarantee the quality and safety of care. The identification, characterization and synthesis of knowledge in this area will seek to be aligned with the objectives and the proposed review question.

The data will be presented in the form of a narrative summary as well as in the form of tables to summarize all the information collected. The tables will contain the main characteristics of the researched supervisory programs.

## 4 Conclusion

With this scoping review, we intend to allow a detailed mapping of the clinical supervision programs that are implemented by the nurse to the caregiver in the community context. We hope that this serves as an incentive for future research in order to contribute to better training the caregiver and to guarantee the quality of care provided. This review is extremely relevant for research, expand the knowledge about clinical supervision in nursing, namely, increase the knowledge about the supervisory programs implemented by the nurse to the caregiver to promote the quality and safety of the care provided.
